# Global trends in physical activity research of attention-deficit/hyperactivity disorder: A scientometric study (2000–2024)

**DOI:** 10.1097/MD.0000000000043794

**Published:** 2025-08-15

**Authors:** Dong Li, Chenmu Li, Jin Yan, Yang Li, Zexi Liu

**Affiliations:** a School of Physical Education and Health, Zhaoqing University, Zhaoqing, China; b School of Physical Education, Guangzhou Sport University, Guangzhou, China; c School of Physical Education and Sports Science, Soochow University, Suzhou, China; d Nanhai Academy of Arts and Technology, Haikou University of Economics, Haikou, China.

**Keywords:** attention-deficit/hyperactivity disorder, bibliometrics, information visualization, physical activity, trends

## Abstract

**Background:**

Increasing evidence shows that physical activity interventions can improve attention-deficit/hyperactivity disorder (ADHD) symptoms. However, understanding of its etiology, treatment, and intervention remains limited. This study systematically analyzes literature from 2000 to 2004 to understand current knowledge, status, and predict future trends, aiming to offer clinicians and researchers a comprehensive perspective and reference for future studies.

**Methods:**

A comprehensive literature search was conducted in the Web of Science Core Collection. Cite Space (version 6.1.R3) and visualization of similarities viewer (version 1.6.10) were employed for data visualization and analysis of publication outputs, collaborative networks, keyword co-occurrence, and co-citation patterns. This bibliometric review was conducted in accordance with the BIBLIO Checklist.

**Results:**

Analysis of 569 articles shows an increasing trend in publications. The United States (279 articles), China (83 articles), and Germany (51 articles) lead in publication volume. Research is concentrated in universities, with Harvard Medical School (17 publications) at the forefront, followed by Natl Taiwan Normal University and University of Wisconsin (14 publications). Authors Chang, Yu-Kai, and Hung, Tsung-Min have the most publications (13 publications). The most cited reference is “DSM-5” with 94 citations. Keywords “ADHD” (172 times), “physical activity” (155 times), and “exercise” (94 times) are the most frequent.

**Conclusion:**

This study highlights the global rise in research on physical activity interventions for ADHD, with the United States leading in output and influence. Physical activity is emphasized as a promising complement or alternative to traditional treatments, offering potential benefits for ADHD symptom management. Key authors, institutions, and collaborations are identified, providing insights into research trends and hotspots. The findings serve as a valuable resource for advancing the understanding and practical application of physical activity interventions. Greater interdisciplinary and regional collaboration is essential for driving innovation and translating research into effective solutions for ADHD management.

## 1. Introduction

Attention-deficit/hyperactivity disorder (ADHD) is an early-onset developmental disorder with a global prevalence of 7.2%.^[1]^ It begins in childhood and persists into adulthood in 30% to 70% of cases,^[2,3]^ affecting approximately 5% of children and 2.5% of adults.^[4,5]^ The prevalence is higher in males than females, with a ratio ranging from 3:1 to 5:1.^[6,7]^ ADHD is characterized by impairments in attention, organization, and/or hyperactivity-impulsivity, with at least 6 symptoms of inattention or hyperactivity/impulsivity lasting for at least 6 months, inconsistent with developmental level, and negatively impacting social and academic activities.^[8]^ ADHD has many comorbidities, including mood disorders, depressive disorders, antisocial personality disorder, and substance use disorders, which may lead to decreased self-esteem, academic underachievement, and reduced occupational success.^[9,10]^ Over the past 20 years, clinicians and researchers have made significant breakthroughs and advancements in technology and methods, enabling better exploration of ADHD and its etiological factors. Despite the increasing amount of literature on the pathology and physiology of ADHD, ongoing exploration and investigation into its inherently complex characteristics are still needed.^[11]^

For the treatment of ADHD patients, medication is one of the therapeutic options. However, in some cases, medications may not meet expectations, be well-tolerated, or be sufficiently effective, and there is potential for misuse and diversion.^[12–14]^ Studies have reported that up to 30% of children respond poorly to medication treatment or cannot tolerate frequent side effects, including insomnia, appetite suppression, growth retardation, and headaches.^[15,16]^ Increasingly, research is leaning towards non-pharmacological treatment strategies to mitigate the potential long-term impacts and associated costs of prolonged use of psychostimulants. Physical activity (PA) is defined as any bodily movement that increases energy expenditure above resting levels, including occupational, sports, recreational, and other activities. Given the prevalence of ADHD and the limitations of traditional treatments, physical activity may be a promising intervention to supplement or replace existing treatment methods.^[17]^ Physical activity has virtually no side effects, is easy to implement, is economically affordable, has limited time constraints, requires minimal parental intervention, and saves parents both financially and mentally.

Physical activity guidelines recommend that children and adolescents engage in 60 minutes of moderate to vigorous physical activity daily.^[18]^ Scientific literature has confirmed the positive effects of physical activity on ADHD patients, demonstrating that physical activity can effectively improve attention, motor skills, and executive function in children with ADHD, without adverse side effects.^[19]^ For young people with ADHD, physical activity may offer particular benefits, especially in areas such as inhibition, selective attention, and cognitive flexibility, promising a bright future. Studies have shown that developing physical activity interventions has a positive impact on the executive function of ADHD patients.^[20]^ As an alternative treatment for ADHD, physical activity holds significant potential for future research.^[21]^ Meta-analyses have indicated that exercise significantly impacts motor skills and executive function in children with ADHD,^[17,22]^ and cognitive involvement in exercise effectively addresses attention issues in school-aged children with ADHD.^[23]^ A recent meta-analysis also showed that aquatic sports and perceptual-motor training exhibit better overall performance, with different physical activity interventions affecting various ADHD indicators differently.^[24]^ In summary, previous research has analyzed the effects of physical activity interventions on ADHD treatment from different perspectives, gaining recognition and providing valuable references for future developments in this field.

In research on other disorders, bibliometrics has made significant contributions. Autism Spectrum Disorder (ASD), one of the most common comorbid conditions with ADHD, has been shown through bibliometric analysis to benefit positively from physical activity interventions. Wang’s bibliometric and visual analysis methods revealed a growing trend in literature focusing on physical activity interventions for ASD and predicted future growth trends.^[25]^ Feng et al conducted a bibliometric analysis of research outcomes related to the impact of physical activity interventions on ASD. By highlighting highly cited articles, active journals, key authors, prominent topics, and research frontiers, their findings may provide valuable guidance for future research directions. In recent years, there has been less bibliometric research on ADHD. However, Liu et al, through a bibliometric analysis of the research themes and development trends of coexisting autism and ADHD, identified overall trends and research hotspots in this field, further proving that interventions for ASD and ADHD will be a focus of future research.^[26]^

Bibliometric analysis employs quantitative methods to study the structure and development of various disciplines. It evaluates and predicts the current status and future trends in research fields through mathematical and statistical measurement methods.^[27,28]^ Bibliometric analysis can quantitatively and qualitatively analyze publications, such as books or journal articles.^[29]^ By analyzing institutions, authors, and keywords within bibliometric analysis, researchers can gain insights into the current status and trends of physical activity interventions in ADHD research.^[30]^ Quantitative analysis of literature can identify differences between research in different regions and the advantages of author collaborations,^[31]^ helping to avoid researcher bias and identify significant research content.^[32]^ The choice of bibliometric analysis is due to its ability to visually and accurately identify the most prominent scientific contributions from countries, institutions, and authors.^[33]^ The number of times an article is cited can indicate the level of interest and engagement researchers have in their published work, which can also reflect the trends needing further development in that field at a particular stage.^[34]^ Thus, employing bibliometric analysis allows for a comprehensive examination of contributions and collaborations from authors, institutions, countries, keywords, and journals,^[29]^ providing a summary of the current status and development trends of a specific disease or research field. This can offer feasible ideas and directions for future research.

Bibliometric analysis has been applied in multiple fields, establishing a solid research foundation. In recent years, research on ASD has gradually increased, while research on ADHD, particularly in the context of physical activity, remains to be further explored. The emerging trends and research hotspots in the field of ADHD and physical activity have yet to be systematically studied. Therefore, this study aims to comprehensively identify the research hotspots, current research directions, and areas needing further improvement from the past 24 years to provide valuable insights for future research. Our study collected articles related to ADHD and physical activity from 2000 to 2024. Using Cite Space and visualization of similarities viewer (VOS viewer), we conducted bibliometric and visual analysis. By examining collaboration networks (including countries, institutions, and authors), annual publication volume, published journals, keyword clustering, keyword co-occurrence, and document co-citation clustering, we aim to understand the research hotspots in different periods and the future development directions in this field.

The main objective of this study is to systematically analyze the content related to physical activity interventions in the treatment of ADHD patients through bibliometric and visual analysis methods, focusing on articles and reviews from the Web of Science (WOS) database. This study aims to understand the structure, current status, and future trends of physical activity interventions in ADHD research. By doing so, it seeks to provide clinicians and researchers with a more comprehensive perspective, uncover new issues, and offer a solid foundation for exploring new research methods.

## 2. Materials and methods

### 2.1. Data search strategy

**Figure 1.**
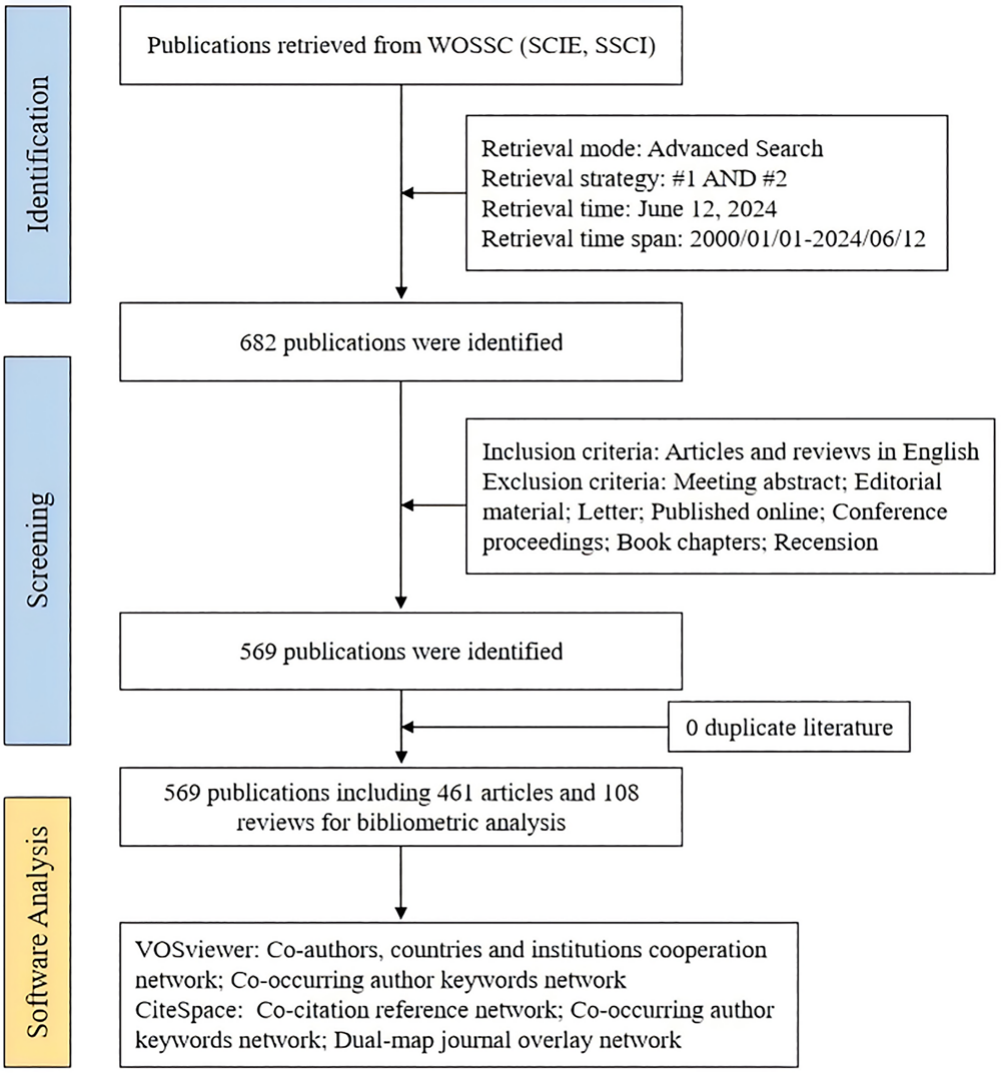
Flow chart of the document selection procedure. SCIE = Science Citation Index Expanded, SSCI = Social Sciences Citation Index, VOS = visualization of similarities viewer, WoSCC =Web of Science Core Collection.

This bibliometric review was conducted in accordance with the BIBLIO Checklist. The data used in this study were primarily obtained from the Web of Science Core Collection (WoSCC). This study uses publicly available data from WOS and therefore does not require a clinical registration number. We conducted a topic search in the Science Citation Index Expanded and Social Sciences Citation Index databases within WOS, all based on the WoSCC database. The retrieval process continued until June 12, 2024, covering the time span from January 1, 2000, to June 12, 2024. The types of literature retrieved were articles and reviews. Choosing appropriate keywords is crucial for bibliometric analysis because it directly affects the research results.^[35]^ Based on the experience from published literature,^[36]^ we performed multiple searches with different keyword combinations, inputting the search terms in the title (TI), keywords (AK), and topic (TS) fields. The specific strategy was as follows: #1 (TI = (“physical activity” OR “physical activities” OR exercise$ OR exercising OR sport$ OR run OR running OR jog OR jogging OR yoga OR “tai chi” OR “tai ji”)) OR (AK = (“physical activity” OR “physical activities” OR exercise$ OR exercising OR sport$ OR run OR running OR jog OR jogging OR yoga OR “tai chi” OR “tai ji”)) AND #2 ((((TS = (Attention Deficit Disorder with Hyperactivity)) OR TS = (ADHD)) OR TS = (Attention Deficit Hyperactivity Disorder)) OR TS = (Hyperkinetic Syndrome)) OR TS = (Attention Deficit Disorder), resulting in a total of 682 documents being retrieved. After manual screening, we included articles and reviews written in English, excluding conference abstracts, editorial materials, letters, online publications, conference proceedings, book chapters, and book reviews. Ultimately, 569 documents were included, comprising 461 articles and 108 reviews (Fig. 1). We extracted data on the following variables: title, publication year, country, institution, journal, author, and keywords.

### 2.2. Data analysis and visualization

The bibliometric method is a quantitative and qualitative combination of author count, word frequency statistics, and citation counts in the literature.^[27,28]^ Bibliometric analysis and data visualization were conducted using Cite Space (version 6.1.R3) and VOS viewer (version 1.6.10). Cite Space, developed by Chen,^[37]^ is widely used to detect emerging trends and visualize knowledge structures in scientific literature. VOS viewer, developed by Van Eck and Waltman,^[38]^ specializes in constructing and visualizing bibliometric networks including co-authorship, co-citation, and keyword co-occurrence networks. Based on the publication time, country, institution, author, and keywords of the literature in the study, we objectively evaluated the current research status of physical activity interventions for ADHD.

Data visualization refers to the use of charts or graphics to clearly and effectively convey and present information.^[39]^ This study used software visualization to present the development trends of physical activity interventions in ADHD. We employed Cite Space and VOS viewer to perform network analyses of collaborating countries, institutions, and authors, as well as to map the data collected from the analysis of keywords and published literature. The time span was from 2000 to 2024, with a time slice of 1 year. The data setting threshold for the top N was 50. We used Pathfinder, pruning sliced networks, pruning merged network slices, and the log-likelihood ratio method for clustering to create a keyword clustering map.

The raw data was exported from the WoSCC database in Bib Tex and CSV formats. The CSV files were imported into VOS viewer to establish national collaboration networks, author collaboration networks, co-citation networks, and term co-occurrence networks.^[38]^ The Bib Tex files were imported into Biblioshiny^[40]^ for analysis, displaying Bradford’s Law model and thematic trends. We used Data Wrapper (https://www.datawrapper.de/ tables, accessed on May 3, 2022) to generate a world map to show the distribution of published articles by region, the output of literature from different countries, and their rankings. We explored the processes, current status, and hotspot issues of physical activity interventions in ADHD research over different periods to identify research priorities and cutting edge themes and to predict future development trends.

## 3. Results

### 3.1. Publication volume and trend analysis

**Figure 2.**
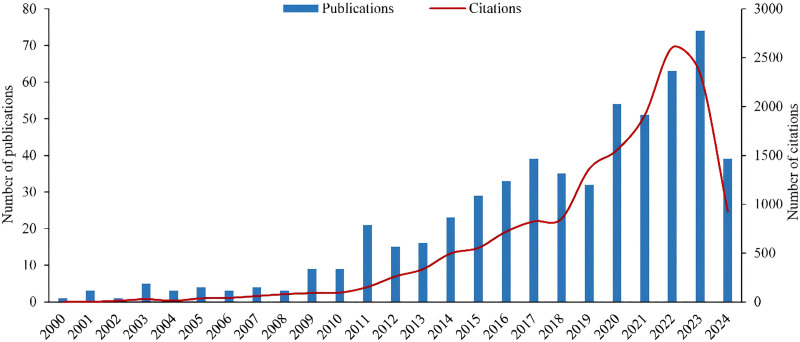
The numbers of documents and citations per year from 2000 to 2024.

Our search retrieved 569 English-language articles on physical activity research related to ADHD published from 2000 to 2024. These articles have been cited a total of 14,809 times, with an average of 26.03 citations per article. The H-index is 64, indicating that at least 64 articles have been cited at least 64 times each. The overall trend shows a general increase in both the number of publications and citations in recent years (Fig. 2). The number of publications peaked in 2023 with 74 articles, and the number of citations peaked in 2022 with nearly 3000 citations. From 2000 to 2008, the number of published articles was relatively low and stable, indicating that research on physical activity in relation to ADHD was in its early stages during this period. In 2011, the number of publications exceeded 20 for the first time, followed by a slight decline in 2012 to 2013. After that, the number of publications in this field showed phased growth, with slight declines in 2014 and 2019, and a peak in 2023. The number of publications in 2024 is low because it only includes the first 6 months of the year. This trend suggests that there has been substantial research in this field in recent years, and it holds significant potential for future research.

**Figure 3.**
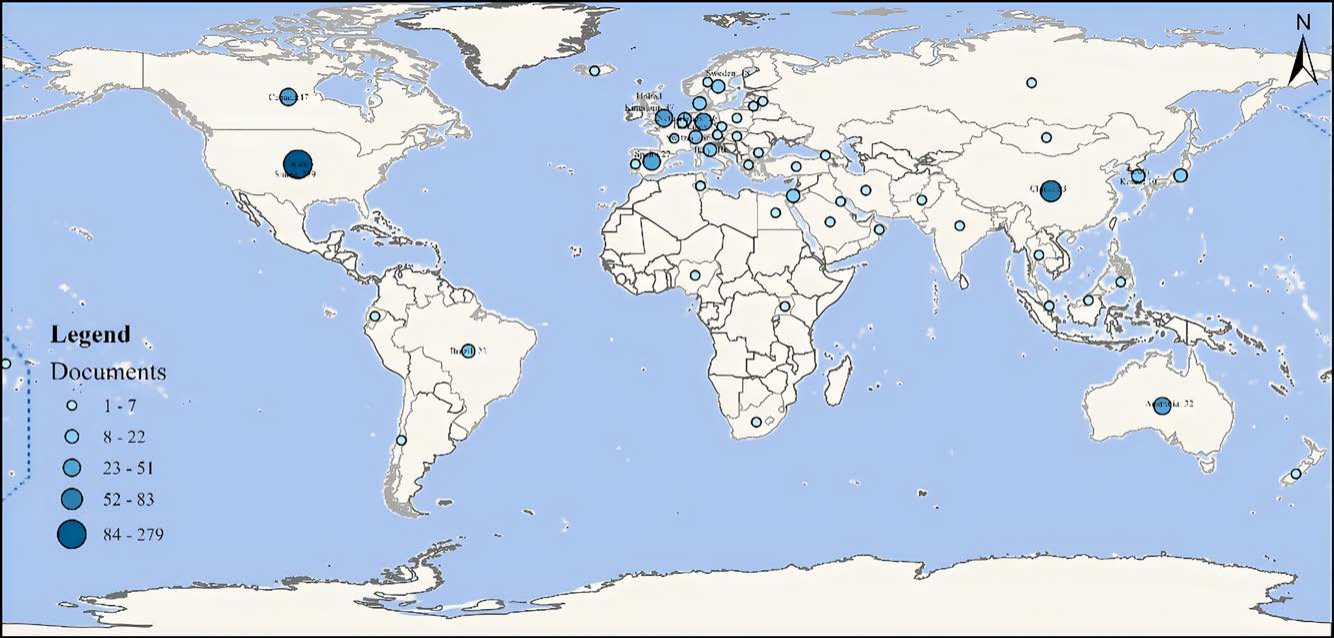
World map based on the total publications of different countries/regions.

Figure 3 visually displays the academic output of different countries and regions in this research field. The United States has the highest number of published articles (279 articles), indicating that academic institutions and researchers in the US have significant influence and contributions in this field. This is followed by China (83 articles), Germany (51 articles), and Canada (47 articles). European countries such as the United Kingdom, Germany, and the Netherlands also have high literature output. The United States, Canada, China, Australia, the United Kingdom, Germany, the Netherlands, and Sweden are major research centers in this field, with high academic output and influence. Other countries and regions, such as Brazil, South Korea, and Japan, also contribute to this field. This world map provides an intuitive understanding of the global academic distribution and the research activity levels of various countries in this field.

### 3.2. Analysis of international collaboration

**Figure 4.**
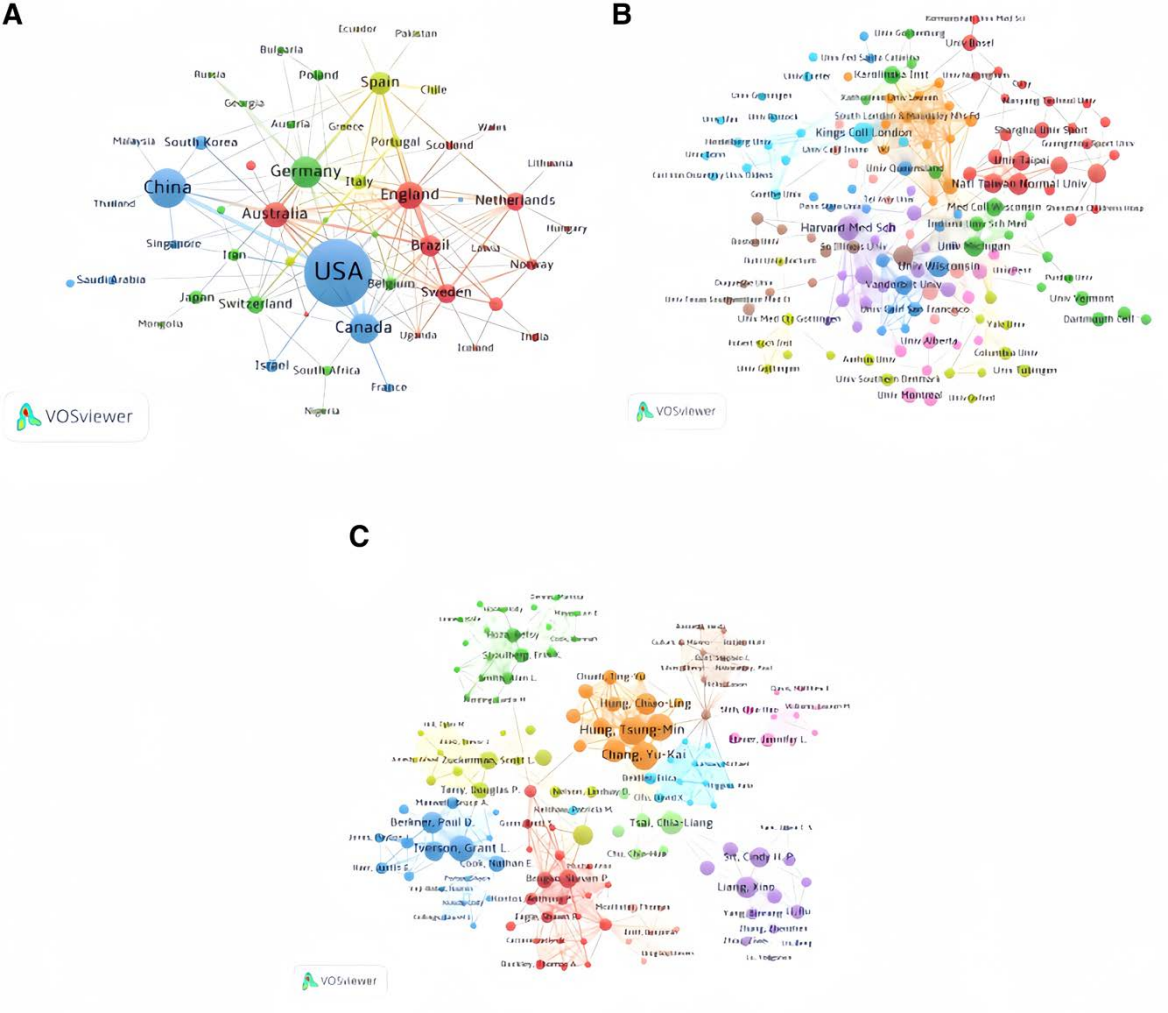
Figure created using VOS viewer. (A) Visualization of countries/regions. (B) Visualization map of research institutions. (C) Visualization map of authors. (Larger nodes indicate that a country, institution, or author appears more frequently in international collaborations. The more lines connecting 2 nodes, the higher the frequency of cooperation between the 2 countries in the same research. Different colored nodes represent different collaboration clusters, which signify distinct groups of collaborative relationships.) VOS = visualization of similarities viewer.

A total of 331 countries/regions have published research related to ADHD and physical activity. Table 1 lists the top 10 countries/regions by publication volume. The United States has the highest number of publications (279 articles), accounting for over 49% of all retrieved articles, but its average citation count (average citation per item [ACI] = 30.8) is not the highest. China is second (83 articles), followed by Germany (51 articles). European countries such as the United Kingdom, Australia, and Spain also have high literature output, with Australia having the highest average citation count (ACI = 52.8). The overall strength of connections between countries is as follows: the United States (total link strength [TLS] = 93), the United Kingdom (TLS = 59), and Australia (TLS = 49). Figure 4A shows extensive international collaboration, with the United States being the most significant collaborator in international research, particularly among the United States, China, the United Kingdom, Germany, Australia, and Canada.

**Table 1:** Top 10 countries/regions published analysis (2000–2024).

Rank	Country	Documents	ACI	TLS
1	USA	279	30.8	93
2	China	83	21.5	32
3	Germany	51	21.6	42
4	Canada	47	33.1	40
5	England	37	32.4	59
6	Australia	32	52.8	49
7	Spain	27	21.6	32
8	Brazil	22	44.0	34
9	Sweden	18	25.9	24
10	Netherlands	16	28	21

TLS ranges from 0 to infinite with higher TLS indicates stronger co-authorship links of a given country/region with other countries/regions.

ACI = average citation per item, TLS = total link strength.

### 3.3. Analysis of institutional collaboration

Among the 569 analyzed articles, 1955 institutions have published research related to ADHD and physical activity. Figure 4B shows a network of 136 international collaborating institutions, with each network having at least 15 coauthored articles. The blue nodes are associated with European institutions, red with Asian institutions, and purple with North American institutions. Major collaborating institutions include Harvard Medical School, the University of Wisconsin, King’s College London, the National Taiwan Normal University, the University of Michigan, the University of Alberta, and Vanderbilt University. The top 10 institutions globally published 129 articles, accounting for 23% of all retrieved articles. Harvard Medical School has the highest number of publications (31 articles after merging duplicates), with a weighted average citation count (ACI = 41.5). The University of North Carolina has the highest average citation count (ACI = 98.5). Other leading institutions include King’s College London (14 articles), National Taiwan Normal University (14 articles), and University of Wisconsin (14 articles; Table 2).

**Table 2:** Top 10 institutions published analysis (2000–2024).

Rank	Institution	Documents	ACI	TLS
1	Harvard Medical School	31	41.5	129
2	Natl Taiwan Normal University	14	25.7	34
3	University Wisconsin	14	41.7	26
4	University N Carolina	13	98.5	20
5	Natl Taiwan Sport University	12	46.1	24
6	University Michigan	12	71.3	40
7	Chinese University Hong Kong	11	10.3	11
8	University Taipei	11	21.8	26
9	Vanderbilt Univ	11	34.5	23

TLS ranges from 0 to infinite with higher TLS indicates stronger co-authorship links of a given institution with other institutions.

ACI = average citation per item, TLS = total link strength.

### 3.4. Analysis of the authors’ collaboration network

A total of 2467 authors have contributed to research on the impact of physical activity on ADHD. Table 3 lists the top 10 authors by the number of publications. Chang Yu-Kai and Hung Tsung-Min have the highest number of publications (13 articles each), followed by Huang Chung-Ju (12 articles) and Iverson Grant L (11 articles). The author with the highest average citation count is Mccrea Michael A (ACI = 49.1).

**Table 3:** Top 10 authors published analysis (2000–2024).

Rank	Author	Documents	ACI	TLS
1	Chang, Yu-Kai	13	46.6	46
2	Hung, Tsung-Min	13	22.8	62
3	Huang, Chung-Ju	12	23.9	49
4	Iverson, Grant L.	11	9.2	50
5	Tsai, Chia-Liang	9	36.6	29
6	Tsai, Yu-Jung	9	18.0	46
7	Berkner, Paul D.	8	9.1	39
8	Hung, Chiao-Ling	8	21.3	38
9	Liang, Xiao	8	10.9	28
10	Mccrea, Michael A.	7	49.1	19

TLS ranges from 0 to infinite with higher TLS indicates stronger collaborative links of a given author with other authors.

ACI = average citation per item, TLS = total link strength.

Figure 4C illustrates the international collaboration among authors. Each node on the graph represents an author, with the size of the circle reflecting the number of publications by that author, and the lines connecting the circles indicating co-authorship relationships. Dense connections between some authors suggest that they have jointly published a significant amount of research in the same or similar fields. Chang Yu-Kai and Hung Tsung-Min have the highest number of publications, and they closely collaborate with Hung Chiao-Ling, potentially focusing on the effects of exercise on executive function and cognitive development.^[41,42]^ Huang Chung-Ju, who has the second highest number of publications and a high TLS value, primarily researches the effects of exercise on psychological and physical health, as well as inhibitory control.^[43,44]^ Iverson Grant L, with the third highest number of publications and the highest TLS value, has made significant contributions to sports medicine, particularly in the diagnosis, treatment, and prevention of concussions, as well as the impact of brain injuries on mental health and related interventions.^[45,46]^

Lines connecting different colored clusters represent cross-cluster collaborations, indicating intersections and integrations between different research fields. By analyzing these collaboration networks, we can identify the main contributors and their collaborators in this field, gaining insights into and predicting the trends of research development.

### 3.5. Analysis of relevant journals

**Figure 5.**
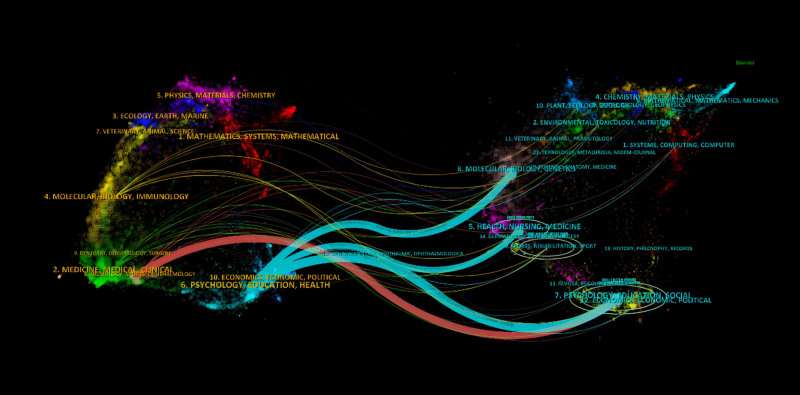
Dual-overlay map of journals.

The dual-map overlay of journals (Fig. 5) shows the citation relationships between journals from different disciplines, reflecting research intersections and academic interactions across various fields. This overlay also describes the distribution of topics within the journals. Thicker connection lines indicate closer citation relationships and more research intersections between disciplines, whereas thinner lines indicate fewer but still present research connections. The left side of the image represents the cited journals, while the right side represents the cited journals. The colored paths illustrate the relationships between them, with 3 main pathways identified between citing and cited journals: from Medicine/Medical/Clinical to Psychology/Education/Health.

### 3.6. Analysis of co-cited references

The top 10 most frequently cited references in the field of physical activity research related to ADHD worldwide are listed in Table 4. The most cited reference is by the American Psychiatric Association,^[47]^ published in 2022 in the “American Psychiatric Association” journal, with 94 citations. This is followed by an article by Pontifex MB et al, published in “J Pediatr-US,” which has been cited 85 times,^[48]^ and an article by Yu-Kai Chang et al, published in “Arch Clin Neuropsych,” cited 77 times.^[49]^ The data shows that from 2012 onwards, the number of citations for articles in this field has significantly increased, indicating that more in-depth and active research has been conducted globally in this area over the past 12 years.

**Table 4:** The top 10 most cited references.

Rank	Citations in the network	Year	First author	Title	Source	DOI
1	94	2022	American Psychiatric Association	Diagnostic and statistical manual of mental disorders: DSM-5	American Psychiatric Association	/
2	85	2013	Pontifex MB	Exercise improves behavioral, neurocognitive, and scholastic performance in children with attention-deficit/hyperactivity disorder	J Pediatr-US	10.1016/j.jpeds.2012.08.036
3	77	2012	Yu-Kai Chang	Effect of acute exercise on executive function in children with attention-deficit hyperactivity disorder	Arch Clin Neuropsych	10.1093/arclin/acr094
4	60	2012	Claudia Verret	A physical activity program improves behavior and cognitive functions in children with ADHD: an exploratory study	J Atten Disord	10.1177/1087054710379735
5	59	2015	Cerrillo-Urbina AJ	The effects of physical exercise in children with attention-deficit hyperactivity disorder: a systematic review and meta-analysis of randomized control trials	Child Care Hlth Dev	10.1111/cch.12255
6	56	2010	Medina JA	Exercise impact on sustained attention of ADHD children, methylphenidate effects	Adhd-Attend Deficit	10.1007/s12402-009-0018-y
7	56	2013	Smith AL	Pilot physical activity intervention reduces severity of ADHD symptoms in young children	J Atten Disord	10.1177/1087054711417395
8	51	2002	Mary Tantillo	The effects of exercise on children with attention-deficit hyperactivity disorder	Med Sci Sports Exerc	10.1097/00005768-200202000-00004
9	45	2015	Choi JW	Aerobic exercise and attention-deficit hyperactivity disorder: brain research	Med Sci Sport Exer	10.1249/MSS.0000000000000373
10	45	2004	Pauline S Jensen	The effects of yoga on the attention and behavior of boys with Attention-Deficit/Hyperactivity Disorder (ADHD)	J Atten Disorder	10.1177/108705470400700403

ADHD = attention-deficit/hyperactivity disorder, DSM-5 = Diagnostic and Statistical Manual of Mental Disorders, Fifth Edition.

**Figure 6.**
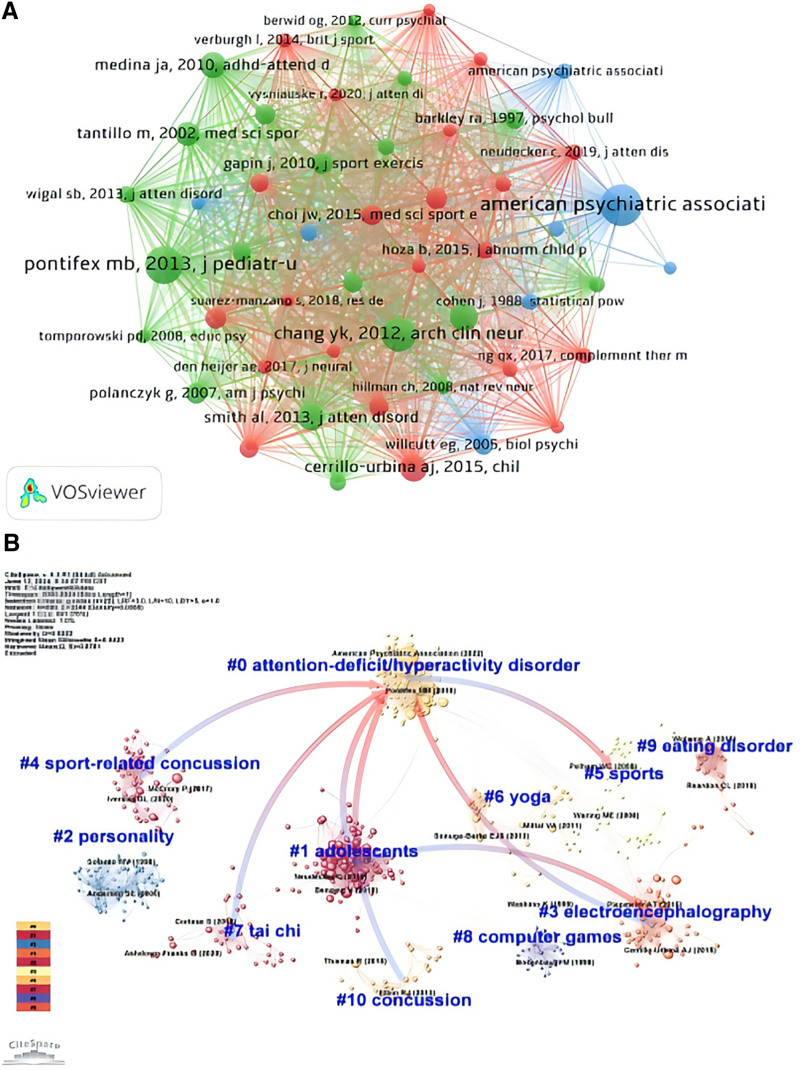
(A) The author’s co-citation network regarding PA research on ASD. (B) Cluster analysis map of co-cited references. (A: The more lines connecting the nodes, the stronger the association between these documents; larger nodes indicate a higher number of citations for that document. The labels next to the nodes display the author, publication year, and journal name of the document.B: Larger nodes in the figure indicate that the document has a higher influence in the field, and thicker connecting lines represent a higher frequency of co-citations.) ASD = autism spectrum disorder, PA = physical activity.

Subsequently, we conducted a co-citation analysis of authors using a co-citation network map (Fig. 6A), which involved 22,891 cited documents. The minimum citation threshold for authors was set at 20, resulting in the inclusion of authors from 72 documents. The nodes representing the American Psychiatric Association and Pontifex MB, 2013, “J Pediatr-US,” are larger, indicating their significant influence and high citation frequency in this field. The node for the “American Psychiatric Association” is particularly large, demonstrating its crucial role in the fields of psychology and psychiatry, and its widespread citation indicates its substantial impact across multiple related research areas. By analyzing the most influential authors, their collaborators, and co-cited documents, we can identify the authors and related literature that have played a beneficial role in advancing research on physical activity and ADHD.

We used Cite Space to perform a cluster analysis of co-cited documents (Fig. 6B). The results show a total of 10 clusters, with the largest cluster being “ADHD.” This cluster includes research on ADHD, indicating its significant influence within the research field. Notable documents such as Pontifex (2013) and the American Psychiatric Association (2022) have high citation rates in their respective clusters, underscoring their important positions in research. Through cluster analysis, we can better understand the relationships and development trends among various research topics.

### 3.7. Keyword analysis

#### 3.7.1. Keyword co-occurrence analysis

**Figure 7.**
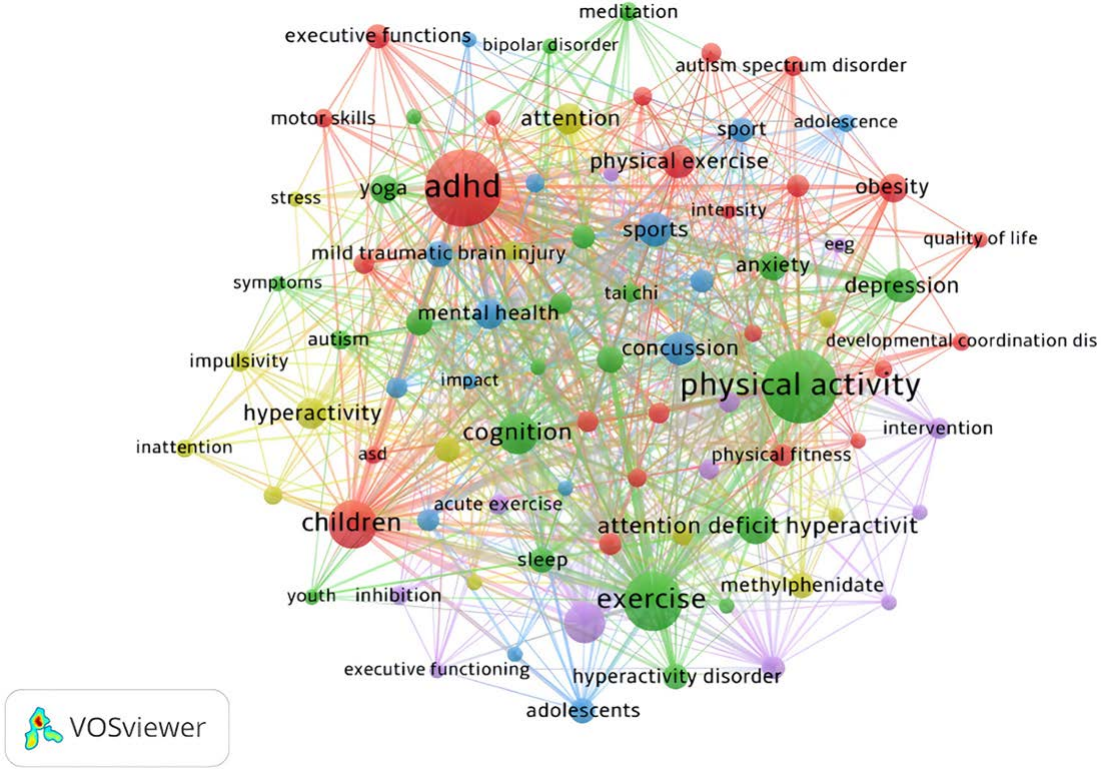
Keyword co-occurrence network map. (The larger the node, the more frequently the keyword appears in the research. The more lines connecting nodes, the stronger the association between these keywords.)

The accuracy and frequency of keywords are 2 critical factors influencing the precision of co-occurrence analysis in identifying research hotspots.^[50]^ This study involved a total of 1416 keywords, setting the minimum occurrence of keywords at 5 times, resulting in the inclusion of 86 keywords. The keyword co-occurrence network map (Fig. 7) displays the keywords and their interrelationships in ADHD and physical activity related research. The network is divided into 5 clusters, represented by different colors: red, green, blue, and purple. For example: The red cluster includes major keywords such as ADHD, yoga, executive functions, and meditation, reflecting the interrelated nature of these topics within their cluster. Larger nodes like ADHD, physical activity, exercise, and children indicate that these keywords are highly important and common in related research. There are many connections between ADHD and keywords such as children, physical activity, and cognition, which are in different colored clusters. This indicates that there are close interconnections and overlaps between research themes from different clusters.

The higher the frequency of a keyword, the more likely it is to appear in the research field, indicating that researchers may be more focused on the directions involving these keywords, which are more likely to become research hotspots.^[25]^ Table 5 lists the top 20 high frequency keywords in this field. In terms of frequency, ADHD has the highest occurrence (172 times), followed by physical activity (155 times) and exercise (94 times). The top 3 keywords have significantly higher frequencies compared to the others, as they are among the key search terms for the data source. Only ADHD and physical activity have frequencies exceeding 100 times, while exercise also has a high frequency, indicating that these 3 keywords attract more attention from scholars in this field. This reflects that research directions related to these 3 keywords are central to the study of physical activity interventions in ADHD. Studies have found that physical activity or exercise interventions for children and adolescents with ADHD can improve core symptoms such as executive function and motor function.^[51]^ Additionally, improving cognitive function, executive function, attention deficits, hyperactivity symptoms, depressive symptoms, concussion conditions, and enhancing mental health in ADHD patients may be important research directions in this field.

**Table 5:** Top 20 high frequency keywords.

Rank	Keywords	Occurrences	Mean (yr)
1	ADHD	172	2018
2	physical activity	155	2019
3	exercise	94	2018
4	children	60	2019
5	cognition	40	2017
6	executive function	40	2019
7	attention-deficit hyperactivity disorder	31	2018
8	depression	27	2018
9	sports	27	2018
10	concussion	26	2019
11	physical exercise	25	2020
12	attention	22	2017
13	hyperactivity	22	2015
14	mental health	21	2020
15	anxiety	19	2018
16	obesity	19	2016
17	yoga	19	2018
18	attention-deficit	16	2021
19	aerobic exercise	15	2018
20	mild traumatic brain injury	15	2018

ADHD = attention-deficit/hyperactivity disorder.

Based on the average year of occurrence for these keywords in the literature, all 20 keywords have appeared within the last decade, with most appearing within the last 6 years. This indicates that recent research trends and hotspots are mainly concentrated in these areas. Additionally, the average year data demonstrates the temporal evolution of these research hotspots, helping us understand the varying levels of attention each keyword has received over different periods.

**Figure 8.**
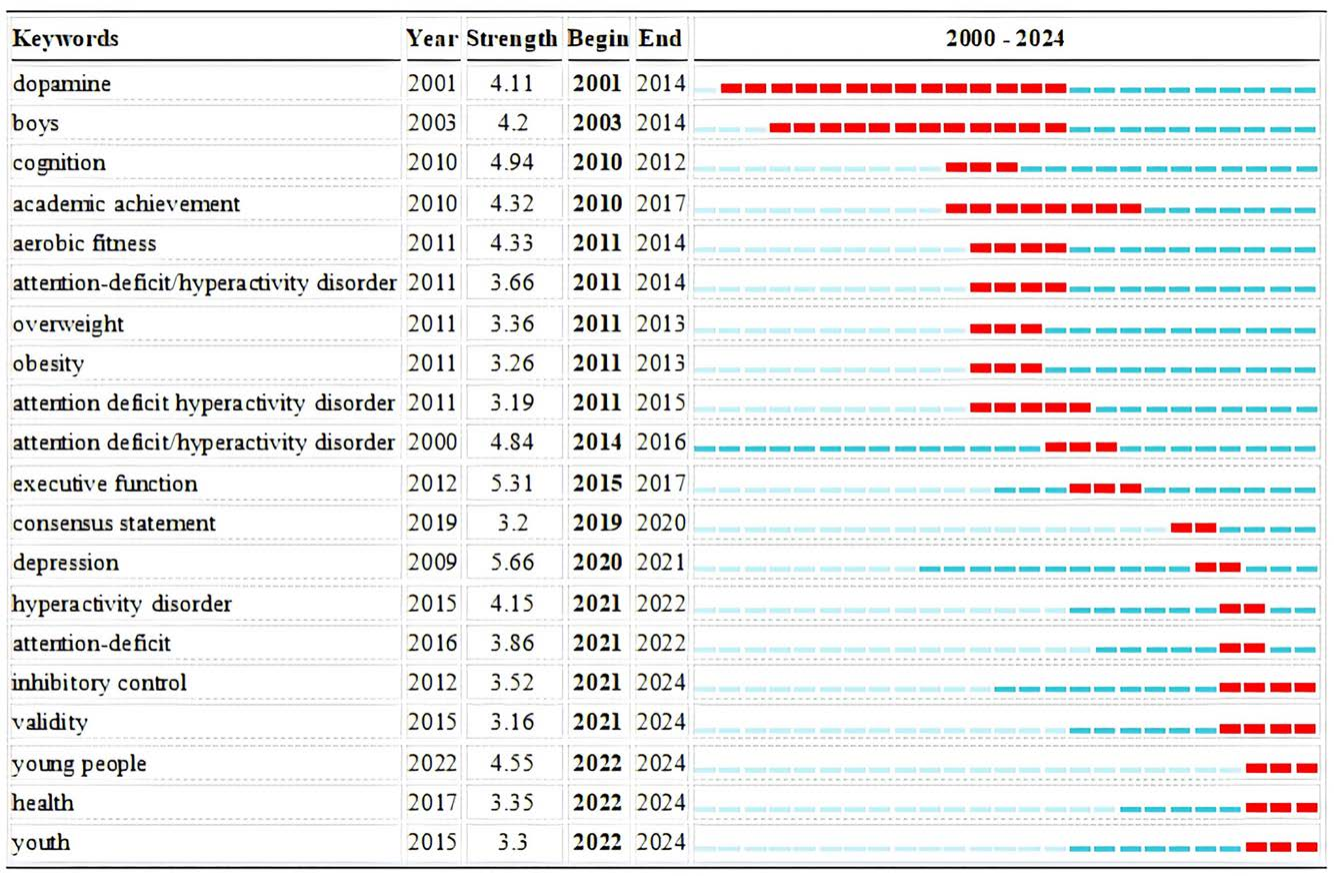
Top 20 keywords with the strongest citation bursts.

By showcasing the burst strength and time period of each keyword, we can understand which research themes were popular and frequently cited during different time frames. The higher the burst strength value, the greater the impact of that keyword during its burst period. Figure 8 displays the top 20 keywords with the strongest citation bursts in the field from the keyword co-occurrence analysis. “Depression” has the strongest citation burst, indicating it has been a key focus of research in this field over the past 20 years. In the past 5 years, “hyperactivity disorder,” “attention-deficit,” “young people,” and “inhibitory control” have shown the strongest citation bursts, suggesting these keywords have recently garnered widespread attention from researchers in this field. “Attention deficit hyperactivity disorder” experienced 2 distinct bursts in 2000 (strength = 4.84) and 2011 (strength = 3.19), indicating significant research interest during these periods. “Consensus Statement” and “Depression” have bursts in the last 5 years, highlighting the importance of research in these areas recently. Additionally, the bursts for “inhibitory control,” “validity,” “young people,” “health,” and “youth” are predicted to end in 2024, indicating that these keywords are likely to remain important research hotspots in the coming years.

#### 3.7.2. Keyword clustering analysis

**Figure 9.**
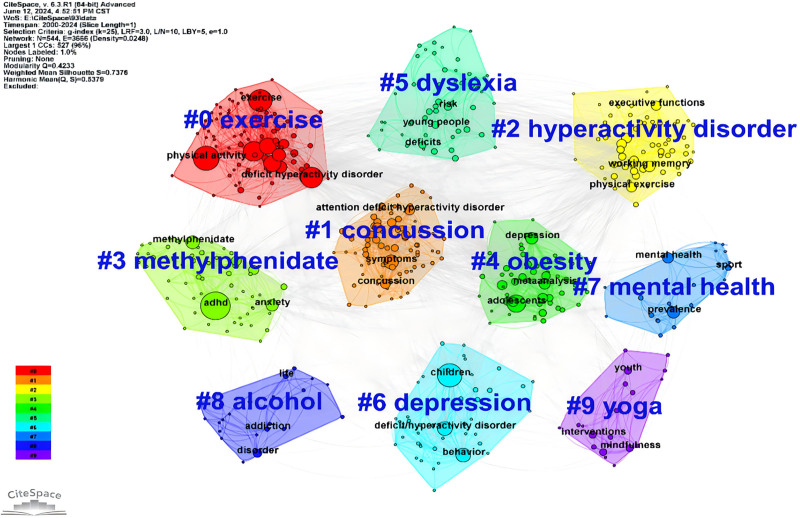
Keyword clustering network map.

The keyword clustering map illustrates the clustering results and timeline of co-cited documents, revealing the main themes and research hotspots in the relevant research field. The clusters are labeled from #0 to #9, and the visualization results are shown in Figure 9. The time distribution of published documents is displayed, ranging from red (earlier) to blue (recent). For example: Cluster #0, labeled “exercise” (red), focuses on research related to exercise. This cluster primarily involves studies connected to exercise, with keywords including “exercise,” “physical activity,” “deficit hyperactivity disorder,” and others. The color gradient from red to blue shows the temporal evolution of research topics, with earlier clusters in red and more recent ones in blue, allowing us to see how research interests and focuses have shifted over time.

The figure reveals that research themes have evolved over different time periods, reflecting the dynamic changes in research hotspots. Early research focused more on fundamental pathology and diagnostic methods, while recent studies have increasingly addressed intervention measures and treatment effects. Cluster #2, labeled “hyperactivity disorder,” shows strong centrality and includes keywords such as “executive function,” “working memory,” and “physical exercise.” This indicates significant interest in the effects of physical activity interventions, which are easier to integrate into the general population and have become the primary target for most researchers studying ADHD physical activity interventions.^[51–53]^ Additionally, keywords like “exercise,” “physical activity,” and “hyperactivity disorder” have attracted considerable attention from the academic community in recent years. For instance: Jeyanthi et al conducted a meta-analysis verifying the positive effects of physical activity on attention, motor skills, and physical health in children with ADHD.^[52]^ Den Heijer’s research provided evidence that physical exercise is a promising alternative or additional treatment option for ADHD patients.^[54]^ Silva’s study showed that a swimming program significantly improved the mental health, cognition, and motor coordination of children with ADHD.^[55]^ Each cluster represents a specific research direction. By analyzing these clusters and their temporal changes, we can better understand the evolution of research themes in this field.

Table 6 lists the relevant information for keyword clusters. Different clusters have interrelated keywords with causal relationships, which help define the research themes. For example, Cluster #0 includes keywords like exercise, executive function, physical activity, concussion, and yoga, indicating that this cluster focuses on how physical activities such as exercise and yoga can improve executive function and address issues like concussions. From the table, we can see that Cluster #0 contains the most keywords (95), suggesting that there has been extensive research on “how physical activities like exercise and yoga can improve executive function and address concussions” for over 13 years. This is followed by Cluster #1 (76 keywords) and Cluster #2 (66 keywords). Cluster #3 has the highest silhouette score (0.839), indicating that the keywords within this cluster are highly cohesive and well-separated from other clusters. In contrast, Cluster #0 has a relatively lower silhouette score (0.692), implying that although it is a large cluster, the cohesion among keywords within the cluster and the separation from other clusters are relatively lower.

**Table 6:** Table of top 10 keyword clusters information.

Cluster ID	Size	Silhouette	Mean (yr)	Label (LLR)
0	95	0.692	2011	exercise; executive function; physical activity; concussion; yoga
1	76	0.67	2015	concussion; mild traumatic brain injury; traumatic brain injury; sports; physical activity
2	66	0.684	2018	hyperactivity disorder; cognitive performance; physical exercise; attention-deficit; inhibitory control
3	62	0.839	2005	methylphenidate; dopamine; wheel running; locomotor activity; anxiety
4	58	0.707	2016	obesity; depression; overweight; sleep; mental disorders
5	57	0.793	2010	dyslexia; reading; cerebellum; phonological awareness; table tennis
6	47	0.716	2008	depression; children; concussion; behavior; deficit/hyperactivity disorder
7	23	0.874	2014	mental health; sports psychiatry; eating disorders; risk factors; adhd
8	22	0.831	2012	alcohol; shell; marijuana; respiratory training; opioids
9	21	0.795	2016	yoga; mindfulness; meditation; tai chi; preschool

Silhouette: A measure of the cohesion and separation of clusters, where values closer to 1 indicate better clustering performance.

Size: The size of the cluster, indicating the number of keywords contained within each cluster.

LL = log-likelihood ratio.

**Figure 10.**
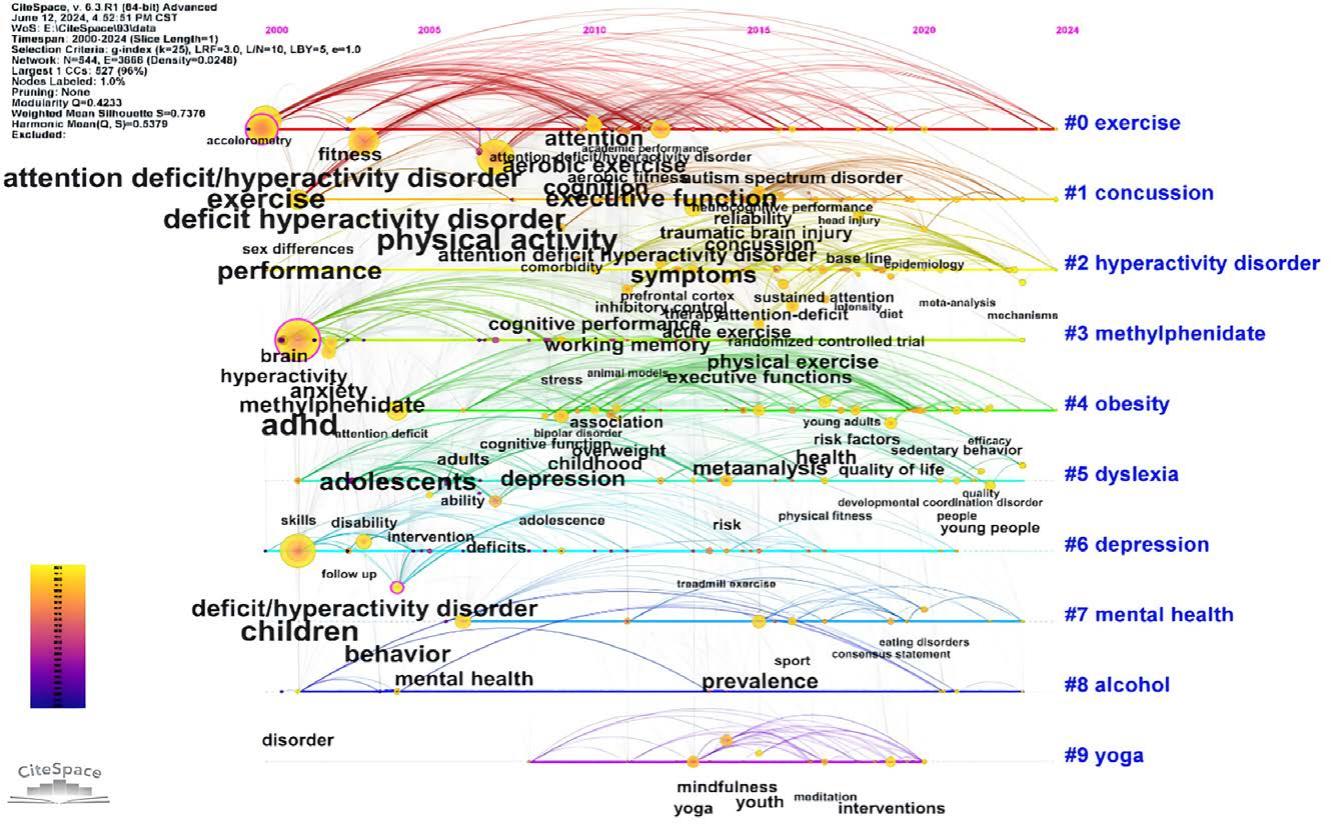
Keyword cluster timeline.

Figure 10 is a timeline of keyword clusters, reflecting the importance and temporal span of specific clusters. The figure displays 10 keyword clusters, with the timeline moving from left to right. The color bar on the left indicates the gradient from 2000 to 2024. From the figure, we can see that research related to “exercise” has continued from 2000 to 2024, illustrating that this area has been a consistent focus of attention. The color changes indicate the evolution of research hotspots over time. For instance, research on “hyperactivity disorder” and “concussion” starts to deepen in color from 2005 onwards, suggesting an increasing focus on these topics after 2005. Some clusters have lighter-colored keywords, indicating that these research areas have emerged as recent hotspots. Since 2010, keywords such as “physical activity,” “aerobic exercise,” “fitness,” “ADHD,” “anxiety,” “obesity,” “weight,” “health,” “cognitive function,” “depression,” “mental health,” “children,” “adolescents,” “yoga,” “mindfulness,” and “interventions” have appeared. This indicates that the exploration and research related to ADHD and physical activity are becoming focal points for researchers.

## 4. Discussion

Our study conducted a bibliometric analysis to evaluate the impact of physical activity interventions on ADHD, aiming to obtain a global overview of the research focus and cutting edge themes in this field. Our results show a gradual increase in the number of published articles from 2000 to the present, with a notable surge in the number and citation rates of articles published between 2019 and 2023. This trend can be attributed to several factors. Firstly, the COVID-19 pandemic, which began in 2020 and lasted until 2022, had a significant impact worldwide. With people largely confined to their homes for work, study, and daily activities, there was a widespread lack of physical activity, which in severe cases could lead to mental health issues. Consequently, researchers increased their focus on the health of vulnerable groups. Secondly, the approval of the Global Action Plan on Physical Activity 2018–2030 (GAPPA) by the World Health Organization (WHO) in 2017 likely played a role. This plan emphasizes the prevention and control of physical inactivity and the promotion of physical activity, and for the first time, includes guidelines for people with disabilities.^[56]^ These developments underscore the equal rights of people with disabilities to health and physical activity, potentially driving broader and higher-quality research in this field.^[57]^

According to the analysis of the national/regional distribution map, research on physical activity related to ADHD shows a global participation pattern. The top 10 countries publishing articles in this field are primarily located in North America, Asia, and Europe. North American and European countries, in particular, are heavily involved and closely collaborate, indicating their leading position in ADHD-related physical activity research. The United States has the highest number of publications, likely due to its advanced research capabilities, a large pool of researchers, and substantial national funding support. Apart from North America and Europe, China has the highest number of publications (83 articles) in Asia. However, the average citation count (ACI = 21.5) for Chinese articles is relatively low. Although China leads in publication volume within Asia, it still lacks high-quality articles. This may be because China’s research in this field started later, with the earliest publication in 2006, 15 years after the United States.^[58]^ It is noteworthy that, among the top 10 countries by publication volume, all are developed countries except China. This indicates that research in this field is significantly lagging in developing countries compared to developed countries. Possible reasons include limited government funding, lower public health awareness, and less developed healthcare systems.^[59]^ Research on high frequency publications and highly cited journals can help us understand the current state of ADHD physical activity research, enrich the theoretical foundation of the field, and improve the quality of related articles. Among the 10 most-cited articles, the “American Psychiatric Association” journal has the highest citation count, followed by “J Pediatr-US” and “Arch Clin Neuropsych.” These 3 journals have demonstrated high-quality and academic value in this field.

In ADHD-related research, the United States is the leading country, with the highest number of cited articles, researchers, and research institutions. The author collaboration network mapping allows us to identify the representative authors in the field of physical activity research related to ADHD. The most prolific author is Chang, Yu-Kai, whose research primarily focuses on the effects of exercise on mental health and cognitive development. His research topics may include exercise interventions for children and adolescents, and the impact of exercise on cognitive disorders such as ADHD and ASD.^[60,61]^ The nodes in the collaboration network are mostly dispersed, with a few densely connected, indicating that only a few authors have close collaborations. For example, Broglio, Steven P., Kontos, Anthony P., and Eagle, Shawn R. show very tight cooperation. Future research could benefit from enhancing interdisciplinary collaborations among scholars to promote innovation and in-depth development in this field. The institution collaboration network mapping reflects the representative institutions globally publishing articles in this field. Harvard Medical School has the highest number of publications and the closest collaborations with other institutions (TLS = 70). Harvard Med Sch, Vanderbilt Univ, and Univ Wisconsin demonstrate the tight cooperation among major US research institutions in medical and health research. Natl Taiwan Normal Univ, Univ Taipei, Shanghai Univ Sport, and Guangzhou Sport Univ highlight the close collaboration of research institutions in sports science and related fields in Asia, particularly Taiwan and mainland China. Strengthening international and regional collaborations in the future could provide more innovative and efficient academic knowledge in this field. Reading highly co-cited references helps quickly grasp important content in the field. The most co-cited document is the American Psychiatric Association’s book “Diagnostic and Statistical Manual of Mental Disorders: DSM-5,” a manual compiled by experts in mental health and related fields. It gathers knowledge about mental disorders to improve conditions for patients, clinicians, researchers, administrators, insurers, and other stakeholders,^[62]^ with ADHD included under the “Neurodevelopmental Disorders” section in the DSM-5. The second most co-cited reference is Pontifex et al’s study, which found that a single bout of moderate-intensity aerobic exercise may positively affect neurocognitive function and inhibitory control in children with ADHD.^[48]^ The third most co-cited reference is Yu-Kai Chang et al’s research, which revealed the positive impact of acute exercise on executive function in children with ADHD.^[49]^ These 3 references are highly recognized and valuable in this field.

By analyzing keywords, we can identify research hotspots in the field of physical activity related to ADHD. We summarized high frequency keywords, performed co-occurrence and clustering analysis, and constructed a timeline for keyword emergence. Our findings indicate that research on physical activity interventions for ADHD primarily focuses on exercise methods and doses related to ADHD, types of physical activities, the child population, cognitive function, and mental health. On one hand, exploring suitable types of physical activities and determining appropriate exercise doses are fundamental for improving the core symptoms of ADHD. This research can help develop the most appropriate intervention plans for ADHD patients. On the other hand, ADHD is considered a highly prevalent neurobehavioral disorder in children,^[63]^ with cognitive function often being impaired. Therefore, research in these areas is crucial for improving ADHD-related symptoms from childhood, thereby reducing the incidence of these symptoms during adolescence and adulthood, improving disease outcomes, and predicting the risk of occurrence.

The research results indicate that the top 3 categories of physical activity related studies on ADHD are exercise and health, adolescent psychology and behavior, and sports and rehabilitation. These 3 categories provide a solid foundation and valuable information for the establishment and development of this field. However, physical activity as an alternative or supplementary intervention plays an important role in ADHD research. Emerging evidence preliminarily shows that physical activity enhances the effectiveness of pharmacological treatment, which is currently the first-line treatment for ADHD.^[64,65]^ The 2018 Physical Activity Guidelines for Americans suggest that acute exercise can be an effective means of promoting brain health.^[66,67]^ Research indicates that brain development in children with ADHD is slower compared to their peers,^[68]^ and these children have lower serum levels of brain-derived neurotrophic factor (BDNF).^[69]^ BDNF is involved in regulating synaptic plasticity and neurogenesis.^[68]^ Existing evidence suggests that sequential aerobic exercise (3 times a week for 45 minutes each, increased to 5 times a week for 60 minutes each over 6 months, with intensity rising from 60% to 90% of maximal oxygen uptake or from 50% to 75% of maximum heart rate) can increase BDNF levels.^[70,71]^ Studies on metabolism indicate that exercise positively affects the neurochemical aspects of the brain, stimulating it and increasing dopamine secretion, which enhances dopamine receptor sensitivity, thereby alleviating ADHD symptoms.^[72,73]^ Physical activity primarily affects the parasympathetic nervous system and vagal stimulation through physiological changes in the cardiovascular system, reducing attention deficits and impulsive behavior.^[74]^ One effective mechanism for reducing ADHD symptoms is through physical exercise, which improves children’s physical motor skills, thereby increasing their willingness to participate in group activities.^[75]^ Additionally, recent findings suggest that a single bout of exercise might be an effective supplementary method for treating ADHD, aligning with the evolving trends and goals of published ADHD treatment guidelines.^[76]^

In comparison with previous scientometric investigations, our study provides a more comprehensive and updated perspective on the interplay between physical activity and ADHD. For instance, Wang et al conducted a bibliometric analysis on physical activity interventions in autism spectrum disorders, using VOS viewer and Cite Space to explore collaboration networks and keyword trends.^[25]^ However, their research did not focus specifically on ADHD and included fewer temporal layers. Feng et al also explored global trends in physical activity research for autism, yet their work primarily focused on publication output and keyword frequency, without addressing institutional or author-level collaboration in-depth.^[77]^ By contrast, our study offers a richer visualization of co-authorship, institutional contributions, and longitudinal keyword evolution across 2 decades, providing more actionable insights for researchers in ADHD-related physical activity fields.

Currently, research on physical activity related to ADHD is still in the developmental stage,^[78]^ and its long-term effects cannot be validated. To explore the long-term impact of physical activity interventions on ADHD patients, it is essential to keep pace with research trends and current hotspots, exploring future research directions to provide the best physical activity intervention methods for ADHD patients. Moreover, future research will continue to focus on improving some core symptoms, psychological health, and physical health of ADHD patients through participation in physical activities. This will help reduce the significant lifelong burden experienced by ADHD patients and their families.

## 5. Limitations

This study used Cite Space (version 6.1.R3) and VOS viewer (version 1.6.10) software to analyze current research on physical activity interventions for ADHD. This analysis provides scholars in the ADHD field with comprehensive insights into the characteristics, hotspots, development trends, and intervention methods. However, this study has some limitations. First, because Cite Space cannot process documents from multiple databases simultaneously, we only retrieved data from the WOS database and did not include other databases. Second, since English remains the most commonly used language for publishing academic literature globally, we only included articles published in English, resulting in a language bias in this study. Lastly, due to the varying quality of the literature, the results regarding the overall structure of this research field may be limited on a global scale.

## 6. Conclusion

This study conducted a bibliometric and visual analysis of research achievements in the field of ADHD and physical activity from 2000 to 2024, revealing global development trends in this area. As a global public health issue, research in the field of physical activity has received increasing attention over the past 5 years. However, the true value of theoretical research results can only be realized when they are successfully translated into educational practice, maximizing the impact of physical activity research on ADHD. Collaboration between countries/regions with different development levels, cooperation between institutions, and partnerships between authors from different countries can collectively advance research in this field. By introducing highly cited articles, high frequency keywords, active journals, key authors, prominent themes, and research hotspots, we predict future development trends and provide a knowledge structure for this field. This offers valuable references for researchers and other stakeholders and serves as a basis for further, more in-depth studies.

## Acknowledgments

We appreciate and thank the reviewers and Associate Editor for their constructive comments and feedback offered during the review process to make this a stronger manuscript.

## Author contributions

**Conceptualization:** Dong Li, Chenmu Li, Zexi Liu.

**Data curation:** Dong Li, Jin Yan.

**Formal analysis:** Dong Li, Chenmu Li, Zexi Liu.

**Investigation:** Dong Li, Jin Yan.

**Methodology:** Dong Li, Chenmu Li, Zexi Liu.

**Project administration:** Dong Li, Chenmu Li, Zexi Liu.

**Resources:** Dong Li, Chenmu Li, Zexi Liu.

**Software:** Dong Li, Zexi Liu.

**Supervision:** Chenmu Li.

**Validation:** Dong Li, Jin Yan, Yang Li.

**Visualization:** Dong Li, Jin Yan, Yang Li.

**Writing – original draft:** Dong Li, Chenmu Li, Jin Yan, Zexi Liu.

**Writing – review & editing:** Dong Li, Chenmu Li, Jin Yan, Yang Li, Zexi Liu.
